# Electrochromic Fabrics with Horizontal Patterning, Enhanced Strength, Comfort, High-Temperature Protection, and Long Coloring Retention Properties for Adaptive Camouflage

**DOI:** 10.3390/molecules30061249

**Published:** 2025-03-11

**Authors:** Jingjing Wang, Haiting Shi, Jixian Gong, Geng Tian, Jinbo Yao

**Affiliations:** 1 School of Textile Science and Engineering, Tiangong University, Tianjin 300387, China; 2 Pharmaceutical Informatics Institute, College of Pharmaceutical Sciences, Zhejiang University, Hangzhou 310058, China

**Keywords:** electrochromic fabrics, hot-pressing technology, cotton fabrics, smart wearables

## Abstract

Electrochromic fabrics (ECFs) can be applied to wearable displays and military camouflage clothing, and they have great potential in developing wearable products. Current ECFs are often bulky, involve complicated processes, and have high production costs. In this study, we report a novel strategy for preparing electrochromic fabrics that require only a three-layer structure: cotton fabric as the substrate, poly(3,4-ethylenedioxythiophene):poly(styrenesulfonate) (PEDOT:PSS) as the electrochromic layer and the electrodes, and an ion-conducting film (ICF) bonded to the fabric by hot pressing. Compared with conventional ECFs, this method does not require the extra preparation of electrode layers on the fabric, as these layers affect the color-changing effect. Hot pressing eliminates the need for a complex sealing process and is more suitable for fabrics with poor wicking effects, which increases the method’s applicability. Cotton fabrics offer the value of biodegradability and are more environmentally friendly. Meanwhile, unlike carbon cloth, the fabric’s color does not interfere with the electrochromic effect. The ICF is non-liquid and can maintain the dryness of the fabric. Additionally, the ICF provides high-temperature protection up to 150 °C. The ECFs exhibit exceptional thinness at 161 µm and a lightweight construction with a 0.03 g/cm^2^ weight. Furthermore, the ECFs exhibit a relatively long sustain time of 115 min without voltage, demonstrating impressive performance. Improved peel strength to 7.11 N is achieved through an improved hot-pressing process. The development strategy for ECFs can also be applied to other electrochromic substances, potentially advancing intelligent applications such as wearable fabrics and military camouflage while promoting rapid progress in electrochromic fabrics.

## 1. Introduction

Smart wearable sensors have been widely developed, including cardiopulmonary [1], humidity [2], temperature [3], knee [4], body motion [5], sweat [6], human breath [7], prosthetics [8], rehabilitation [9], and so on. The fast market growth has pushed forward the evolution of textile-based [10,11,12] and skin-like (epidermal) [13,14] wearable technologies. Fabrics serve as the perfect base for adaptable electronic devices [15,16]. Fabrics rank as the most commonly used material in everyday life. These entities possess attributes like ease of breathing, a soft texture, and adaptability to align with the body’s contours. Hence, textiles may be regarded as additional skin for human use [17,18].

Current technologies for realizing intelligent discoloration mainly include electrochromic, photochromic [19], thermochromic [20], and so on. Among them, electrochromic (EC) technology is the preferred solution for the preparation of smart color change due to its excellent electrochemical controllability, low energy consumption and rich color change [21,22,23,24,25]. Typically, electrochromic substances encompass transition metal oxides, coordination polymers of transition metals, violet rosettes, and organic conjugated polymers, among others [26,27,28,29]. ECDs (electrochromic devices) have received increasing attention due to their potential in the field of flexible electronics [30,31,32], and they are now widely used in information displays, sensors, smart windows, camouflage coatings, anti-glare mirrors, and displays [33,34,35,36]. In most cases, however, electrochromic devices are constructed on rigid substrates. Electronic textiles are important in microelectronics and their application on textile substrates is also being actively explored. Given that the substrate constitutes the majority of the device mass, textiles are superior to conventional electronics because of their lower weight and biodegradability. The ability to replace glass or plastic with textile material is a critical step. Meanwhile, with their rough and porous nature, fibers are excellent for promoting capillary transport and uptake. In a word, as a substrate, fibers offer advantages over commonly used substrates such as poly (ethylene terephthalate) (PET) or glasses as they are porous, wicking, and biodegradable.

Flexibility and wearability can be achieved by combining EC technology with flexible substrate materials [37]. The ability of electrochromic materials enables individuals to generate their unique creations and directly incorporate these into their apparel. As soldiers’ environments change, their clothing may constantly adapt to the most suitable color scheme [38]. Textiles that incorporate electrochromic materials hold great potential for displaying and transmitting visual information and signals, from camouflage to visual interfaces [39,40].

However, current research on electrochromic fabrics still faces many challenges. Some researchers use PET plastic and fabric integration, but the integrated device cannot meet the softness of the fabric itself; some researchers directly integrate the yarn, resulting in the yarn being too thick and hard, which damages the softness of the fabric itself when woven into it [41,42]. The use of liquid electrolyte 1-butyl-3-methylimidazolium tetrafluoroborate directly affects the dry properties of fabrics [43]. The carbon cloth itself has a dark color, which can interfere with the color-changing effect [44]. The electrolyte was filled into the middle nylon fabric by vacuum filtration [45]; which is a time-intensive process, often taking tens of minutes to several hours and requiring multiple cycles to achieve sufficient filling, thus significantly reducing production efficiency. Moreover, this method is not suitable for high-viscosity ion-conductive membranes or intricate fabric structures, as it is prone to incomplete filling or pore blockage. To overcome the above difficulties, this article uses polyurethane electrolyte and integrates it with fabric through hot-pressing technology. Polyurethane has a certain degree of viscosity and good fastness when combined with the fabric. This technique is compatible with fabrics that have limited core absorption efficiency, while successfully preserving their original softness and dryness. The chosen fabric is pure white cotton, distinguished from carbon cloth, providing a clearer representation of the differences that occur during the color change. Additionally, the use of a parallel structure aids in color comparison while minimizing the complexity of experimental procedures. Cotton fabric has different biodegradability from polyester fabric and nylon fabric, and it is more environmentally friendly, which will be beneficial for the widespread promotion of electrochromic fabrics.

## 2. Results and Discussion

### 2.1. Performance Analysis of Electrochromic Fabrics

**Figure 1:**
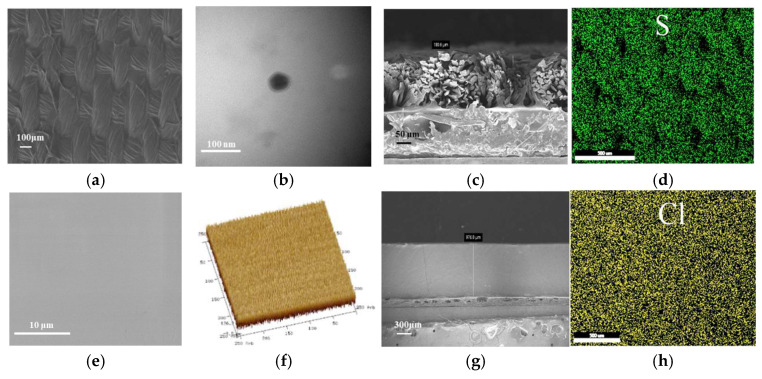
(**a**) The SEM image of layer-treated fabrics, (**b**) the TEM image of the core-shell structure PEDOT:PSS, (**c**) the cross section of layer-treated fabrics, (**d**) the EDS mapping of the S element of layer-treated fabrics, (**e**) the SEM image of an ICF, (**f**) the surface roughness of ICF, (**g**) the cross section of ICF, (**h**) the EDS mapping of the Cl element.

Figure 1a shows the surface of the electrochromic layer; the uniform distribution of PEDOT:PSS can be observed on the surface of the treated fabric. It is shown in Figure 1e that the surface of the ICF is distributed uniformly. It is observed from Figure 1b that the hydrophobic property of PEDOT makes it localized at the core of the macromolecule, with PSS as a hydrophilic part surrounded at the edge of the macromolecule. As mentioned above, the PEDOT:PSS has a core-shell structure. PSS has three main roles in this conductive polymer. PSS acts as a dispersant of PEDOT and enables the dispersibility of the PEDOT in water. The backbone of PSS served as a template during the EDOT polymerization; a polyanion polystyrene sulfonate provides sulfonate ions that exert a stabilizing effect on the PEDOT cations. Figure 1f shows that the WPU-LiClO_4_ membranes displayed a roughness value of Ra = 45.3 nm. The roughness of pure WPU film is 7.8 nm. It follows that WPU-LiClO_4_ membranes have a similar flatness to pure WPU film. The thickness of the layer-treated fabrics was 180.6 µm (Figure 1c). The thickness of the WPU-LiClO_4_ membrane is determined to be 976 µm (Figure 1g). After the hot-pressed layer-treated fabrics with the WPU-LiClO_4_ membrane, the overall thickness is 720 µm, indicating that cotton fabrics and membrane were compounded together excellently. S elements are the characteristic elements of PEDOT:PSS. Cl elements are the characteristic element of LiClO_4_. Figure 1d,h show that PEDOT:PSS was uniformly distributed on the pattern surface of the cotton fabric and LiClO_4_ was well dispersed in the WPU.

**Figure 2:**
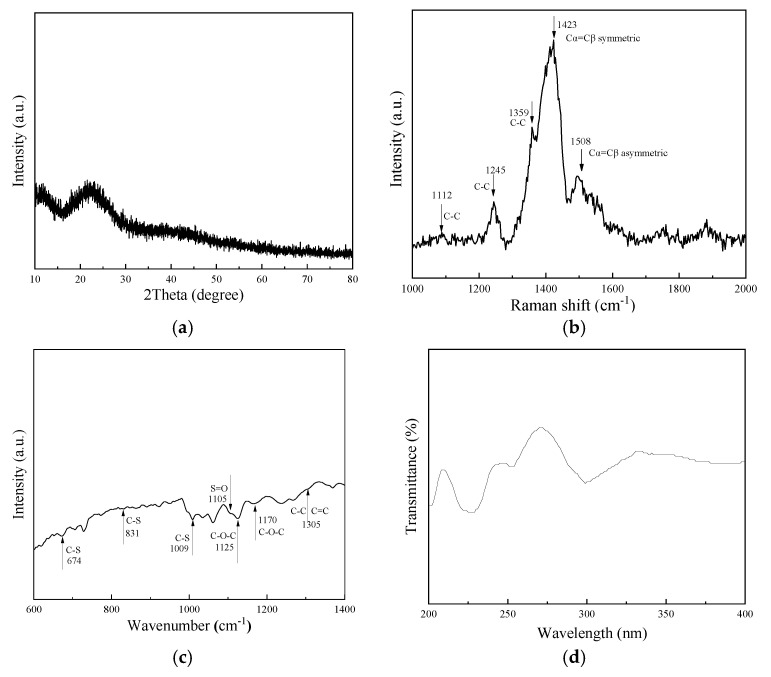
Performance of PEDOT:PSS color changing layer: (**a**) the XRD of the samples, (**b**) the Raman images of the samples, (**c**) the FTIR spectra of the samples, (**d**) the UV band of the samples.

As shown in Figure 2a, the XRD pattern of PEDOT:PSS showed an amorphous characteristic, with a broad peak at 2θ = 10 and 22. In the Raman map in Figure 2b, the characteristic peaks at 1112 cm^−1^, 1245 cm^−1^, and 1359 cm^−1^ correspond to the C-C plane bending, stretching vibration, and extensional deformation, respectively. There are two structures in the PEDOT. One is the 1423 cm^−1^ of the Cα=Cβ symmetric stretching vibration, and the other is the Cα=Cβ asymmetric stretching vibration of thiophene rings in the 1508 cm^−1^. This exceptional structural configuration was advantageous for the conductivity of the PEDOT:PSS film [45,46,47]. Figure 2c: FT-IR spectrum of PEDOT:PSS film. The absorption peaks at 1305 cm^−1^ were ascribed to the stretching vibrations of the quinoidal structure of the thiophene ring’s C=C and C-C. The bands of vibration at 1170 and 1125 cm^−1^ corresponded to the methylenedioxy group’s C-O-C stretching vibrations. The thiophene ring’s C-S band extending emerged in the absorption bands at 674, 831, and 1009 cm^−1^. Additionally, the 1105 cm^−1^ bands are assigned to the S=O symmetric stretching modes in PSS [47,48]. UV-vis transmittance spectra of PEDOT:PSS are shown in Figure 2d. There are some strong or weak absorption peaks in the UV region (250–350 nm). The K-band of energy levels of polymer molecules with π-bonds is shown. These peaks represent the electron transition from π electron orbitals to the antibonding π* orbitals, which can be expressed simply as π→π*. Strong absorption was observed at 300 nm, which indicated the multiple π-π conjugation existence of PEDOT. Polythiophenes are conducting polymer doped (p-type) [49].

**Figure 3:**
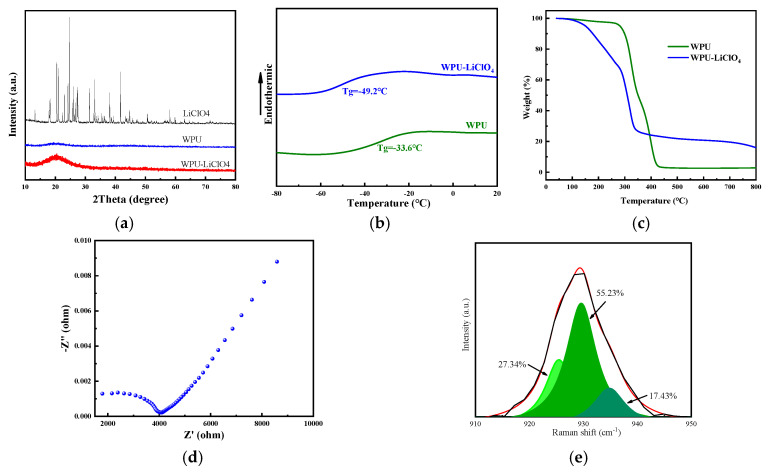
Performance of ICF: (**a**) the XRD of the sample, (**b**) the DSC of the sample, (**c**) the TGA of the sample, (**d**) the impedance curve of the sample, (**e**) the Raman images of the sample.

In Figure 3a, the XRD results show that high-crystalline LiClO_4_ is used, but based on diffraction peaks with broader widths, WPU exhibits an amorphous structure. WPU-LiClO_4_ follows the structure of WPU more closely. The XRD patterns of WPU-LiCLO_4_ do not exhibit any readily discernible diffraction peak of ion pairs, suggesting that the WPU may have dissolved the LiClO_4_. Furthermore, the less strong and wide diffraction peak at 2θ = 19.6° indicates that the addition of salt significantly generates an amorphous structure in the polymer matrix. This might be explained by the fact that Li+ coordinates with the N-H and C=O groups to break the hydrogen bonds between the hard segments. Additionally, the formation of an evident amorphous system structure increases with increasing salt concentration. Figure 3b shows the DSC curves of the WPU and WPU-LiClO_4_ films. The glass transition temperature (Tg) decreased from −33.6 °C to −49.2 °C. The addition of LiClO_4_ suggests that LiClO_4_ disrupted the extent of hydrogen bonding between the hard and soft segments of WPU, which makes more soft segments divorced from the bond by hydrogen bonding. Therefore, the Tg becomes smaller. Thermogravimetric Analysis (TGA) curves of WPU-LiClO_4_-based electrolytes are shown in Figure 3c. The T_,5_*_%_* decomposition temperature is decreased from 282 °C to 150 °C with the addition of LiClO_4_. The contact between the lithium salt and the WPU’s hard segment, which weakens the hydrogen bonding connection between the two segments, might account for this behavior. The WPU-LiClO_4_ films’ T_,5%_ values were found to be over 150 °C, suggesting that it is safe above this temperature. In comparison to the traditional PEO-based electrolyte, which is typically below 80 °C, the working temperature is greater. The electrochemical impedance plot results are shown in Figure 3d, from which it can be seen that the ionic conductivity (Equation (1)) of the WPU-LiClO_4_ electrolyte is 0.244 × 10^−4^ S cm^−1^ at room temperature.

From Figure 3e, it can be seen that the ClO_4_^−^ anion symmetrical telescopic vibration of the 930 cm^−1^ peak from the Raman analysis of the electrolytes can be divided into three peaks, indicating that there are three forms in which the ionic group exists. In the one located at 925 cm^−1^, 27.34% of ClO_4_^−^ ions were present in the form of free ions. In the other peak, with 55.23%, located at 929 cm^−1^, ClO_4_^−^ ions were present in the form of ion pairs. The last, with peaks at 934 cm^−1^, 17.43% of ClO_4_^−^ ions were present in the form of ion aggregates.

**Figure 4:**
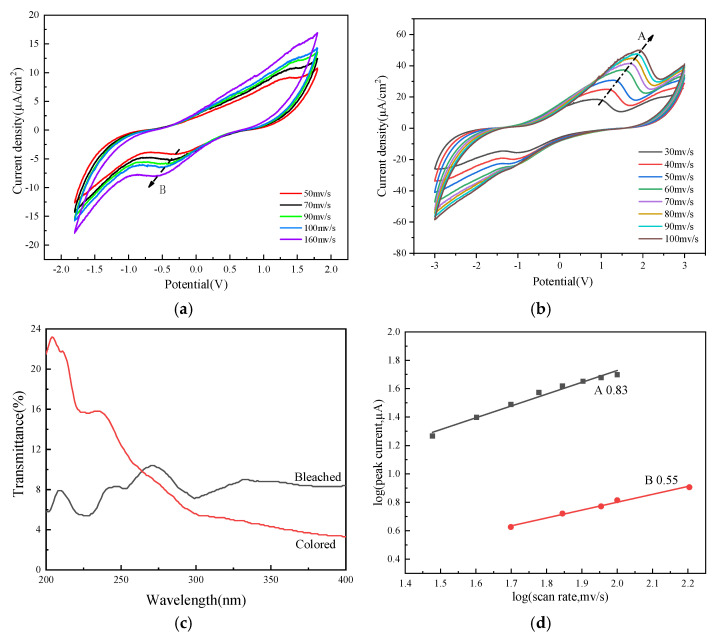
The properties of electrochromic fabrics: (**a**) the reductive peaks of the CV Curve, (**b**) the oxidation peak of the CV Curve, (**c**) the UV band results, (**d**) the b value results.

As shown in Figure 4c, in the doped status, the formation of polarons and bipolarons, a delivery model of alternating resonance of single and double bonds in the molecular chain direction is formed. The extent of delocalization is greater due to the branched chains on the thiophene ring [48]. Different scanning speeds were employed to conduct CV tests for the ECFS (Figure 4a,b). As the scanning speed escalates, reduction peaks migrate towards lower potentials while oxidation peaks shift to higher potentials. This occurs because the redox process may reach the whole active surface area when scanning slowly. However, due to time limitations at elevated scanning speeds, diffusion restricts the mobility of Li^+^. When conducting the redox reaction, only the outermost active layer is utilized, leading to the polarization of the electrode, namely, redox peak shifts. The relationship between maximum current (i) and scanning speed (*v*) underwent analysis under the presumption that the current follows a power–law correlation (Equation (2)). Specifically, when b = 1, the resulting current originates from electrochemical reactions controlled by the surface, known as capacitiveness; when b = 0.5, it is governed by a semi-infinite process of linear diffusion. Figure 4d shows the log(i) and log(v) graphs for the anode peak A1 and cathode peak B1, which are approximately straight-lined, suggesting that the electrochemical reaction is reversible. After the curves were fitted linearly, it became apparent that the b-values (Equation (2)) for the peak currents of the anode and cathode were 0.83 and 0.55, in that order; this suggests that the majority of the anode peak currents were regulated by the surface, while the cathode peak currents were primarily governed by semi-influential linear diffusion.

The polymer PEDOT:PSS is a cathodically coloring material. PEDOT:PSS exhibits a bleaching effect when exposed to positive voltages and a color-changing effect under negative voltages. An electric field is applied, causing the doping and de-doping of ions of electrolytes, which leads to the interaction between the polymeric components that can modify the band structure of the material, hence achieving the electrochromic effect. The electrochromic properties of PEDOT:PSS film were tested in the LiClO_4_ electrolyte. The mechanism is expressed as below:

$${\mathrm{PEDOT}:\mathrm{PSS}:\mathrm{ClO}}_{4}{+2e}^{-}{+\mathrm{Li}}^{+}↔\mathrm{Li}\;\mathrm{PEDOT}:\mathrm{PSS}+{{\mathrm{ClO}}_{4}}^{-}$$

**Figure 5:**
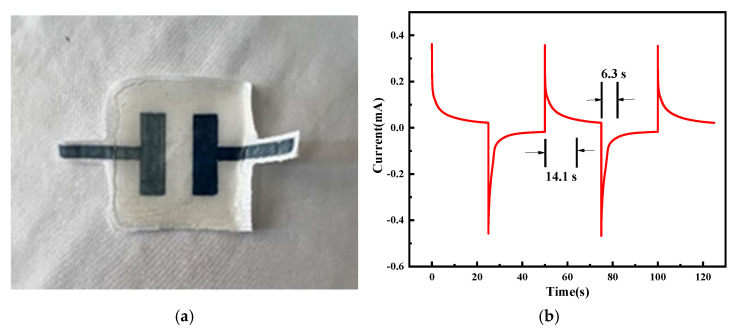
The electrochromic fabric’s electrochromic properties: (**a**) the electrochromic fabric in its colored (**right**) and colorless (**left**) states, (**b**) current-time reaction curve ranging from voltage −1 V to +1 V.

Figure 5a illustrates light blue in its fading state and navy blue in its colored state, each at varying voltage levels. Reaction time serves as a crucial metric to assess the ECFs’ efficiency, defined as the amount of time needed for a 90% shift in the overall current density. The data in Figure 5b illustrate that the electrochromic fabric’s color adjustment period, amidst varying potential levels ranging from −1.0 V to +1.0 V, approximates 14.1 s for coloration and 6.3 s for bleaching purposes.

**Figure 6:**
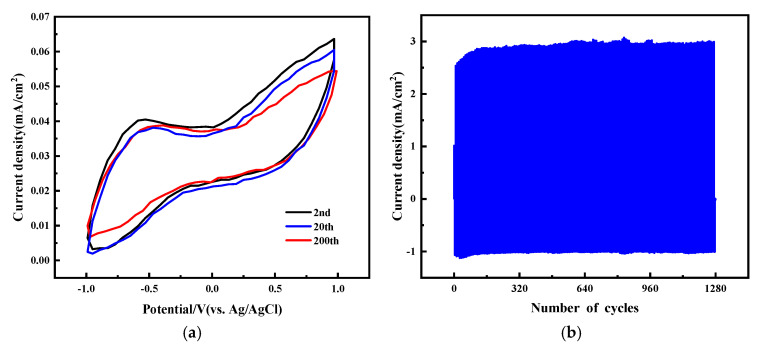
Stability of electrochromic fabrics: (**a**) the CV assessments results, (**b**) the CA assessments results.

Cycle stability is a critical factor in determining the practical applicability of electrochromic fabric. CV and CA assessments are conducted to check the stability of the cycles in electrochromic cotton fabrics. Figure 6a illustrates that the capacity for ion storage drops by merely 2.9% following 200 cycles (Equation (3)). Figure 6b illustrates that, within a potential range of −1.0 V to 1.0 V, there was no marked decline in the electrical current density of the electrochromic fabric following 1280 cycles.

**Figure 7:**
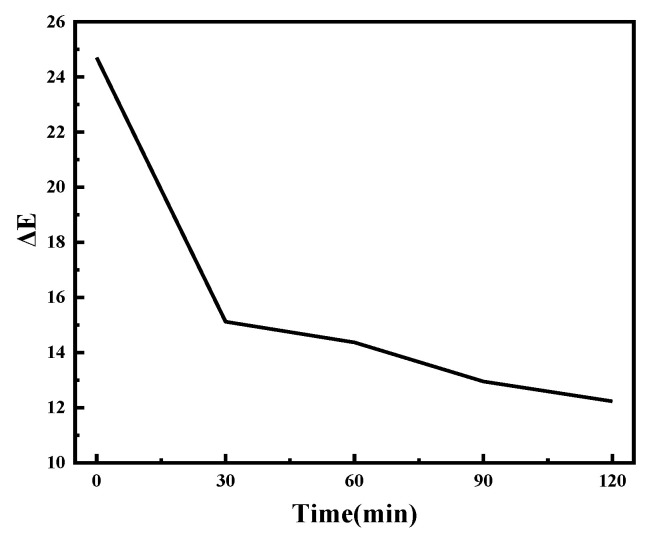
Time diagrams for the optical memory.

When ΔE values fall below 1, the colors are imperceptible to human vision, while ΔE values between 1 and 10 are perceptible, though the colors might be similar. Differentiating two colors requires an ΔE value of 10 or higher. The average color contrast of the ECFs in both their colorful and colorless states can be computed. The L a b coordinates of the pixels in their colored form are (22.92, 2.21, −23.07), which shift to (44.26, −1.48, −11.20) in the colorless state, leading to a color disparity of ΔE = 24.70 (Equation (4)). For the human eye, there is a shift in color from a very light blue shade to a dark blue color, as one can also see in Figure 5a. The optical memory refers to the capability of an electrochromic material to sustain its coloration even when voltage is removed, so the electrochromic device is more energy-efficient than other display devices. Optical properties can be maintained without energy expenditure and are useful for displaying static images or words. Table 1 and Figure 7 show that the time to decay to 50% of the ΔE original value is 115 min.

### 2.2. Enhanced Peel Strength by Refining the Hot-Pressing Process

**Figure 8:**
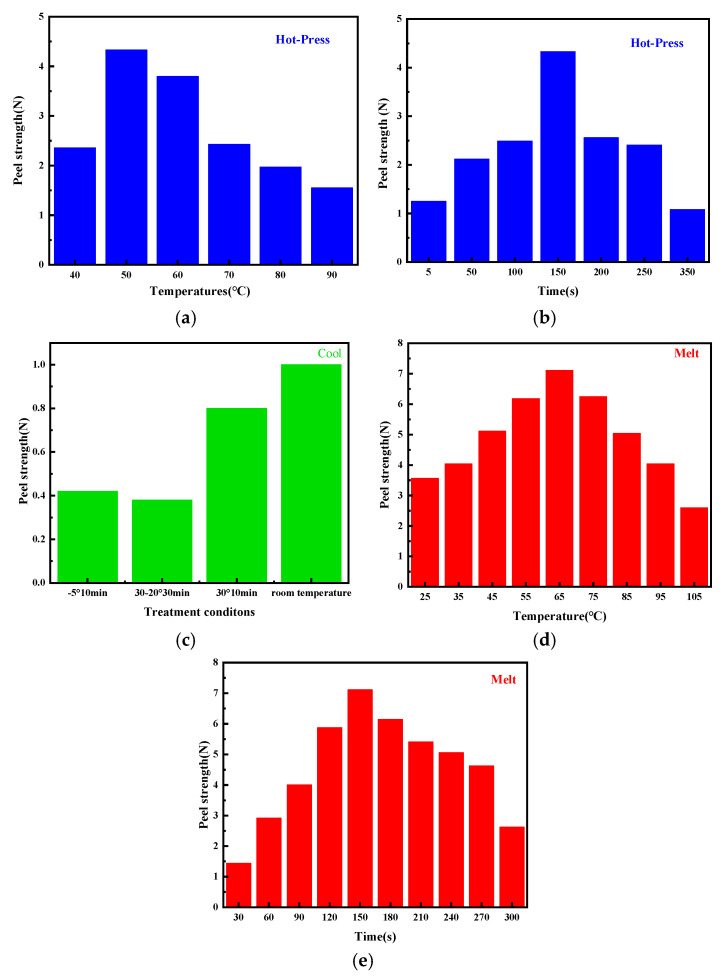
The peel strength results of the prepared fiber under different conditions:(**a**) different hot-press temperature stages, (**b**) different hot-press time stages, (**c**) different cool stages, (**d**) different melt temperature stages, (**e**) different melt time stages.

The data presented in Table 2 and Table 3 and Figure 8a,b indicate that the liquid hot melt adhesive fibers either form adhesive bonds or are tightly bonded to the fibers through mechanical force. The low temperature causes the molten liquid hot melt adhesive to move in reverse. Excessive temperature can damage the strength of the membrane, and experimental results indicate that the optimal temperature is 50 °C. Too short a time results in insufficient adhesion between the membrane and fibers, while too long a time leads to reverse adsorption of liquid hot melt adhesive. The experimental results indicate that the optimal time is 150 s.

Table 4 and Figure 8c illustrate the impact of cooling solidification on peel strength. The bonding effect between the hot melted adhesive and the fibers in the film, if the cooling stage is rushed to cool down, will greatly reduce the bonding strength of the already formed bonding effect. So slow cooling at room temperature has a good effect.

Table 5 and Table 6, along with Figure 8d,e, indicate that the heated area of the electrolyte membrane melts. The liquid adhesive glue causes the interface molecules to come into contact with each other through wetting. if the temperature is too low, the film cannot melt; if the temperature is excessively high, it may result in damage to the film’s strength. Experimental results show that the optimal temperature is 65 °C. The liquid melted adhesive molecules cross the interface and enter the interior of the fiber to form a diffusion interface zone, achieving adsorption equilibrium through molecular motion. The contact between liquid melt adhesive and the fiber surface and the establishment of adsorption equilibrium may cause reverse motion if the time is too long. If the time is too short, the transport power is limited, so the optimal time is 150 s.

Hydrogen bonding serves as a crucial mechanism for the adhesion between polyurethane and cotton fabrics. Polyurethane molecules feature polar groups, including amino groups (-NH) and hydroxyl groups (-OH), while cotton fibers are primarily made up of cellulose, which is rich in hydroxyl groups as well. The formation of these hydrogen bonds significantly enhances the adhesion and stability of the bond between polyurethane and cotton fabrics, thereby improving the overall strength of the composite materials.

In summary, the optimal hot-pressing process is to first melt at 65 °C for 150 s, then hot press at 50 °C for 150 s before removing, and finally cool at room temperature. From the chart, temperature has a significant impact on peel strength. If the hot-pressing temperature is too low, it cannot bond well with the fabric, and if the temperature is too high, it will damage the strength of the film. Under optimal conditions, the peel strength can reach 7.11 N, meeting the requirement of FZ/T81007-2022 [50] for peel strength greater than or equal to 6 N.

### 2.3. Boosted Comfort by Fabricating Ultra-Thin Electrochromic Fabrics

**Figure 9:**
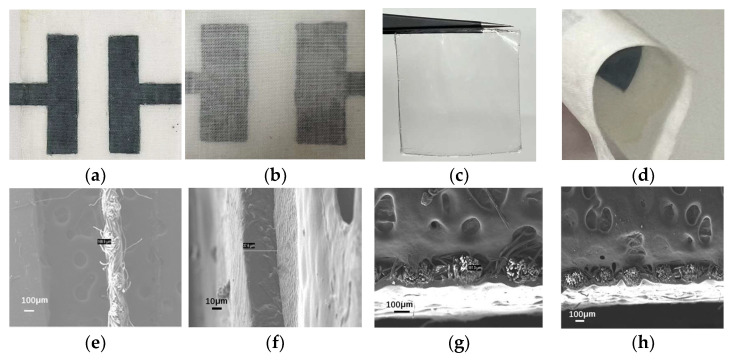
(**a**) Front view of electrochromic fabric, (**b**) back view of electrochromic fabric, (**c**) physical image of electrolyte film, (**d**) bending effect image of electrochromic fabric, (**e**) thickness image of cotton fabric, (**f**) thickness map of electrolyte membrane, (**g**) thickness map of fabric and electrolyte after hot pressing, (**h**) effect map of fabric and electrolyte after hot pressing.

Figure 9 illustrates that the electrochromic fabric is remarkably ultra-thin (a), dry (b), and has good flexibility (d), while the electrolyte film is ultra-thin (c). From Figure 9 (e,f), the thickness of the fabric body is 198.9 microns, the thickness of the ICF is 37.6 microns, and the thickness of the electrolyte membrane and fabric after hot-pressing treatment is 161 microns (g), which is smaller than the thickness of the fabric body. This indicates that the hot-pressing treatment can make the fabric tighter.

## 3. Experimental Section

### 3.1. Materials

Waterborne polyurethane was produced by Anhui dowell Huatai new materials Co., Ltd. (Hefei, China). Lithium perchlorate was produced by Shanghai Aladdin Biochemical Technology Co., Ltd. (Shanghai, China). Conductive PEDOT: PSS ink was acquired from Shanghai OE chemicals Co., Ltd. (Shanghai, China). Propylene carbonate (PC) was bought from Shanghai Titan Scientific Co., Ltd. (Shanghai, China). cotton fabric was purchased from Lutai Textile Co., Ltd. (Zibo, China). All chemicals were used without further purification.

### 3.2. Electrochromic Cotton Fabrics Assembly

**Figure 10:**
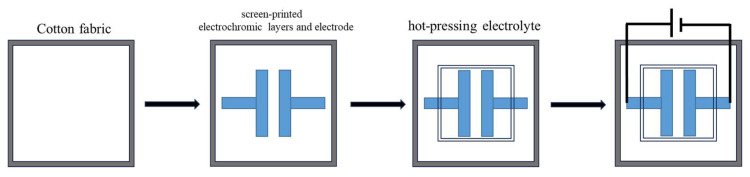
Schematic representation for preparation of electrochromic cotton fabrics.

The fabrication process for constructing lateral fabric-based ECDs is outlined in the schematic in Figure 10. First, cotton fabric was washed with deionized water, acetone, and ethanol to remove impurities. Then, two PEDOT:PSS (1 cm × 3 cm) are screen-printed on the fabric as discolored regions and two PEDOT:PSS lines (0.5 cm × 3 cm) are printed as connectors when connected to a power source (Step 2 in Figure 10). The ICF used here was made as follows: LiCLO_4_ was dissolved in PC at a concentration of 2.6 mol/L, 3 g was taken out, 5 g of waterborne polyurethane (WPU) solution was added, the mixture was stirred for 10 min, then poured in a PTFE mold and oven-dried at 55 °C. The ICF was hot-pressed over the fabric at 40 °C for 30 min in an ambient atmosphere (Step 3 in Figure 10). And finally, the power supply was turned on.

The device’s charge equilibrium, guaranteeing swift and sharp contrast shifts even at minimal operational voltages, was managed by maintaining consistent PEDOT:PSS coverage on both PEDOT:PSS pixel surfaces. Although these fabrics use basic pattern designs, more intricate patterns are readily achieved.

### 3.3. Sample Characterization

A scanning electron microscope (SEM, crossbeam 550, Carl Zeiss, Oberkochen, Germany) was used to characterize the morphology and thickness of the sample; the detailed structure was further characterized by a transmission electron microscope (TEM, Hitachi H7650, Hitachi, Tokyo, Japan); atomic forcemicroscopy (AFM, Icon, Brook, Brooksville, FL, USA) was used to analyze the nanometer-scale surface roughness. The element distribution and percentage were analyzed with an energy-dispersive spectrometer (EDS, EDAX, Carl Zeiss, Oberkochen, Germany); the chemical structures were analyzed by a Fourier transform infrared (FTIR, Nicolet iS50, Thermo Fisher Scientific, Waltham, MA, USA) spectrometer; the crystal structure was analyzed using an X-ray diffractometer (D8 ADVANCE, BRUKER, San Jose, CA, USA); the molecular structure was detected by Raman spectroscopy (XploRA PLUS, Horiba, Tokyo, Japan); the UV-vis spectrum was characterized by a UV-vis-NIR spectrophotometer (UH4150, Hitachi, Tokyo, Japan); thermal analysis methods such as thermogravimetry (TG, 209 F3 Tarsus, NETZSCH GmbH, Hanau, Germany) and differential scanning calorimetry (DSC, 200F3, NETZSCH GmbH, Hanau, Germany) were utilized to discuss thermal properties of polymeric materials. The testing program for DSC involves a temperature range from −80 °C to 200 °C, with a heating rate of 10 °C/min. For TG analysis, the temperature was increased from room temperature to 800 °C, also at a heating rate of 10 °C/min. All electrochemical measurements were carried out under the same ambient conditions using an electrochemical workstation (CHI-660E, Shanghai, China). An intelligent constant temperature heating table from Dongguan Bangyuan Electronics Co., Ltd. (Dongguan, China) was employed to ensure stable thermal conditions for the bonding of ICF film to fabric, complemented by a heat transfer printing machine from Dongguan Gaoshang Machinery Co., Ltd (Dongguan, China) that exerted pressure and heat to facilitate proper adhesion.

To determine the membrane’s ionic conductance, the formula used was as follows:

(1) σ=d/R×S

where σ (S cm^−1^) represents the ionic conductivity; d (cm) denotes the membrane’s thickness; S (cm^2^) is the membrane’s surface area; R (Ω) signifies the point where impedance spectroscopy crosses the abscissa.

The power–law relationship is shown below:

(2) $$i=av^{b}$$

Here, *a* and *b* are calibrated to ensure a proper fit. i represents the maximum electrical flow, while v denotes the scanning velocity.

The capacity for storing ions was delineated through the CV curves, calculated in the following way:

(3) C=(∫Idv)/(2vsV)

wherein *C* represents the particular capacitance of the substance, the symbol *I* denotes the current value of the reaction, *V* represents the value of voltage, *v* denotes the scan rate of cyclic voltammetry, and *s* is the area of the electrodes’ surface.

Optical contrast (Δ*E*) was calculated from CIE Lab color values according to the following equation:

(4) $$∆E=\sqrt{∆L^{2}+∆a^{2}+∆b^{2}}$$

Data were acquired by two-point measurements between −1.0 V and +1.0 V. CIE Lab color difference values indicate the difference in the electrochromic layer in the initial and end colored state. A digital camera was used to take photographs of the polymer electrochromic device at high resolution. The color was quantified using CIE Lab color coordinates [51]. The L component represents the lightness of a color, where 100 is diffuse white and 0 is black, the a is the red/green component, and the b quantifies the yellow/blue component.

Peel strength: the Instron 5969 universal strength machine (Instron) was utilized as per the FZ/T80007.1-2023 [52] testing standard, the sample is cut into 25 mm × 150 mm and pulled at a rate of 100 mm/min. Check whether it meets the requirements according to FZ/T81007-2022 [50].

## 4. Conclusions

In this study, it has been demonstrated that a new type of electrochromic device utilizing cotton fabric as the electrochromic base substrates can be fabricated. Cotton fabrics differ from glass, plastic, polyester fabrics, and nylon fabrics in the value of their biodegradability and in being more environmentally friendly. The ionic conductivity of the prepared electrolyte was 2.44 × 10^−5^ S cm^−1^ at room temperature. 27.34% of ClO_4_^−^ ions were present in the form of free ions, 55.23% of ClO_4_^−^ ions were present in the form of ion pairs, and 17.43% of ClO_4_^−^ ions were present in the form of ion aggregates. In the colored status of PEDOT:PSS, the formation of polarons and bipolarons, a delivery model of alternating resonance of single and double bonds in the molecular chain direction is formed. Most of the anode peak currents of the ECFs were controlled by the surface; most of the cathode peak currents were controlled by semi-infinite linear diffusion. Currently, the challenges faced by electrochromic fabric technology revolve around several key aspects. The first is material stability, which requires that the fabric maintains its performance across multiple cycles. Experimental results indicate no marked decline in electrical current density after 1280 cycles, with only a 2.9% drop observed after 200 cyclic voltammetry (CV) cycles, demonstrating the stable performance of the electrochromic fabric. Second, the high production costs present a considerable barrier to commercial application. Our production method is fast, efficient, and fabric-friendly (non-acidic), eliminating the need for separate electrodes and minimizing manufacturing requirements. Additionally, it does not require encapsulation, significantly reducing production costs. Third, achieving a balance between technological implementation and user comfort is crucial. This study highlights the innovative features of the electrochromic fabrics, including ultra-thin thickness (161 µm), low weight (0.03 g/cm^2^), softness, noticeable color contrast (ΔE = 24.70), high-temperature resistance (up to 150 °C), long color retention (115 min), and rapid response times (14.1 s for coloring and 6.3 s for bleaching). In summary, the developed fabric enhances wearing comfort while ensuring optimal electrochromic performance. Fourth, many electrochromic materials raise environmental concerns. However, the cotton fabric selected for this research is biodegradable, thus improving its eco-friendly attributes. Fifth, it is essential to address the compatibility issue between electronic components and textile materials. The method proposed in this article effectively resolves this challenge without the need for additional electrodes. Through these efforts, we aim to find a balance between meeting technical requirements and ensuring user experience and environmental sustainability in the research and application of electrochromic fabrics. The developed electrochromic fabrics are highly competitive in the market and can be applied across various fields, including smart clothing, wearable health monitoring devices, dynamic displays, and furniture decoration. Therefore, the developed electrochromic fabric not only showcases technological innovation but also holds significant market potential and social value in practical applications.

## Figures and Tables

**Table 1:** Change in color lightness over time.

Time/Min	L	a	b
colored	22.92	2.21	−23.07
30	30.86	1.31	−17.63
60	31.19	1.07	−16.60
90	32.57	0.96	−16.21
120	33.26	0.75	−16.05
faded	44.26	−1.48	−11.20

**Table 2:** The influence of different hot-press temperature stages of the hot-pressing process on peel strength.

Melt	Hot-Press	Cool	180° Peeling Strength (N)	Peel Off State
Room temperature	40° 150 s	Room temperature	2.36	film fracture
	50° 150 s		4.33	film fracture
	60° 150 s		3.80	film fracture
	70° 150 s		2.43	film fracture
	80° 150 s		1.97	film fracture
	90° 150 s		1.55	film fracture

**Table 3:** The influence of different hot-press time stages of the hot-pressing process on peel strength.

Melt	Hot-Press	Cool	180° Peeling Strength (N)	Peel Off State
Room temperature	50° 5 s	Room temperature	1.25	complete peeling
	50° 50 s		2.12	film fracture
	50° 100 s		2.49	film fracture
	50° 150 s		4.33	film fracture
	50° 200 s		2.56	film fracture
	50° 250 s		2.41	complete peeling
	50° 350 s		1.08	complete peeling

**Table 4:** The influence of different cool stages of the hot-pressing process on peel strength.

Melt	Hot-Press	Cool	180° Peeling Strength (N)	Peel Off State
120° 150 s	50° 150 s	−5° 10 min	0.42	complete peeling
		30-20° 30 min	0.38	complete peeling
		30° 10 min	0.8	complete peeling
		Room temperature	1.00	film fracture

**Table 5:** The influence of different melt temperature stages of the hot-pressing process on peel strength.

Melt	Hot-Press	Cool	180° Peeling Strength (N)	Peel Off State
25° 150 s	50° 150 s	Room temperature	3.56	complete peeling
35° 150 s			4.04	complete peeling
45° 150 s			5.11	film fracture
55° 150 s			6.18	film fracture
65° 150 s			7.11	film fracture
75° 150 s			6.25	film fracture
85° 150 s			5.04	complete peeling
95° 150 s			4.04	complete peeling
105° 150 s			2.60	complete peeling

**Table 6:** The influence of different melt time stages of the hot-pressing process on peel strength.

Melt	Hot-Press	Cool	180° Peeling Strength (N)	Peel Off State
65° 30 s	50° 150 s	Room temperature	1.44	film fracture
65° 60 s			2.92	film fracture
65° 90 s			4.01	film fracture
65° 120 s			5.88	film fracture
65° 150 s			7.11	film fracture
65° 180 s			6.14	film fracture
65° 210 s			5.41	complete peeling
65° 240 s			5.06	complete peeling
65° 270 s			4.63	complete peeling
65° 300 s			2.63	complete peeling

## Data Availability

Data will be made available on request.

## References

[1] Zhang H., Wang Z., Teng C., Kumar S., Li X., Min R. (2023). Wearable Cardiorespiratory Sensor for Real-Time Monitoring with Smartphone Integration. IEEE Trans. Instrum. Meas..

[2] Peng Y., Long Z., Liang S., Zhong T., Chen M., Xing L., Xue X. (2023). A battery-free music-driven humidity sensor for intelligent wearable sensing system in smart diaper. Smart Mater. Struct..

[3] Usman M., Jamhour N., Hettinger J., Xue W. (2023). Smart wearable flexible temperature sensor with compensation against bending and stretching effects. Sens. Actuators A Phys..

[4] Faisal A.I., Majumder S., Scott R., Mondal T., Cowan D., Deen M.J. (2020). A Simple, Low-Cost Multi-Sensor-Based Smart Wearable Knee Monitoring System. IEEE Sens. J..

[5] Homayounfar S.Z., Andrew T.L. (2020). Wearable Sensors for Monitoring Human Motion: A Review on Mechanisms, Materials, and Challenges. SLAS Technol..

[6] Xu W., Hong L., Zheng J., Li M., Hua Y., Zhao X. (2023). Wearable Smart Sensor System for Monitoring and Intelligent Prediction of Sodium Ions in Human Perspiration. IEEE Internet Things J..

[7] Li Y., Liu C., Zou H., Che L., Sun P., Yan J., Liu W., Xu Z., Yang W., Dong L. (2023). Integrated wearable smart sensor system for real-time multi-parameter respiration health monitoring. Cell Rep. Phys. Sci..

[8] Cutrone A., Micera S. (2019). Implantable Neural Interfaces and Wearable Tactile Systems for Bidirectional Neuroprosthetics Systems. Adv. Healthc. Mater..

[9] Patel S., Park H., Bonato P., Chan L., Rodgers M. (2012). A review of wearable sensors and systems with application in rehabilitation. J. NeuroEng. Rehabil..

[10] Saikia M.J. (2023). Smart Fabric and E-Textile Sensor Technology for Wearables to Measure High Pressure. IEEE Trans. Instrum. Meas..

[11] Ma J., Shen L., Jiang Y., Ma H., Lv F., Liu J., Su Y., Zhu N. (2022). Wearable Self-Powered Smart Sensors for Portable Nutrition Monitoring. Anal. Chem..

[12] Ha J.-H., Jeong Y., Ahn J., Hwang S., Jeon S., Kim D., Ko J., Kang B., Jung Y., Choi J. (2023). A wearable colorimetric sweat pH sensor-based smart textile for health state diagnosis. Mater. Horiz..

[13] Chen F., Zhuang Q., Ding Y., Zhang C., Song X., Chen Z., Zhang Y., Mei Q., Zhao X., Huang Q. (2023). Wet-Adaptive Electronic Skin. Adv. Mater..

[14] Xu C., Song Y., Sempionatto J.R., Solomon S.A., Yu Y., Nyein H.Y.Y., Tay R.Y., Li J., Heng W., Min J. (2024). A physicochemical-sensing electronic skin for stress response monitoring. Nat. Electron..

[15] Xing T., He A., Huang Z., Luo Y., Zhang Y., Wang M., Shi Z., Ke G., Bai J., Zhao S. (2023). Silk-based flexible electronics and smart wearable Textiles: Progress and beyond. Chem. Eng. J..

[16] Peng H., Li H., Tao G., Xia L., Xu W., Zhai T. (2023). Smart Textile Optoelectronics for Human-Interfaced Logic Systems. Adv. Funct. Mater..

[17] Chatterjee K., Tabor J., Ghosh T.K. (2019). Electrically Conductive Coatings for Fiber-Based E-Textiles. Fibers.

[18] Meng X., Wu Q. (2021). Design of an Interactive Device Based on e-Textile Material. HCI International 2021—Late Breaking Papers: Multimodality, eXtended Reality, and Artificial Intelligence.

[19] Xiao Y., Shen M., Li J., Wang H., Sun H., He Y., Huang R., Yu T., Huang W. (2024). Thermally Activated Photochromism: Realizing Temperature-Gated Triphenylethylene Photochromic Materials. Adv. Funct. Mater..

[20] Liu B., Rasines Mazo A., Gurr P.A., Qiao G.G. (2020). Reversible Nontoxic Thermochromic Microcapsules. ACS Appl. Mater. Interfaces.

[21] Gu C., Jia A.-B., Zhang Y.-M., Zhang S.X.-A. (2022). Emerging Electrochromic Materials and Devices for Future Displays. Chem. Rev..

[22] Poh W.C., Eh A.L., Wu W., Guo X., Lee P.S. (2022). Rapidly Photocurable Solid-State Poly(ionic liquid) Ionogels For Thermally Robust and Flexible Electrochromic Devices. Adv. Mater..

[23] Yin L., Cao M., Kim K.N., Lin M., Moon J.-M., Sempionatto J.R., Yu J., Liu R., Wicker C., Trifonov A. (2022). A stretchable epidermal sweat sensing platform with an integrated printed battery and electrochromic display. Nat. Electron..

[24] Fan H., Wei W., Hou C., Zhang Q., Li Y., Li K., Wang H. (2023). Wearable electrochromic materials and devices: From visible to infrared modulation. J. Mater. Chem. C.

[25] Ling Y., Li L., Liu J., Li K., Hou C., Zhang Q., Li Y., Wang H. (2023). Air-Working Electrochromic Artificial Muscles. Adv. Mater..

[26] Jo M.-H., Kim K.-H., Ahn H.-J. (2022). P-doped carbon quantum dot graft-functionalized amorphous WO3 for stable and flexible electrochromic energy-storage devices. Chem. Eng. J..

[27] Banasz R., Wałęsa-Chorab M. (2019). Polymeric complexes of transition metal ions as electrochromic materials: Synthesis and properties. Coord. Chem. Rev..

[28] Lu H.-C., Kao S.-Y., Yu H.-F., Chang T.-H., Kung C.-W., Ho K.-C. (2016). Achieving Low-Energy Driven Viologens-Based Electrochromic Devices Utilizing Polymeric Ionic Liquids. ACS Appl. Mater. Interfaces.

[29] Lo C.K., Shen D.E., Reynolds J.R. (2019). Fine-Tuning the Color Hue of π-Conjugated Black-to-Clear Electrochromic Random Copolymers. Macromolecules.

[30] Valiūnienė A., Virbickas P., Medvikytė G., Ramanavičius A. (2019). Urea Biosensor Based on Electrochromic Properties of Prussian Blue. Electroanalysis.

[31] Xiao S., Zhang Y., Ma L., Zhao S., Wu N., Xiao D. (2020). Easy-to-make sulfonatoalkyl viologen/sodium carboxymethylcellulose hydrogel-based electrochromic devices with high coloration efficiency, fast response and excellent cycling stability. Dye. Pigment..

[32] Lang A. (2020). Electrochromic Devices Incorporating Conjugated Polymers and Cellulose: New Opportunities for Organic Electronics.

[33] Wibowo A.F., Han J.W., Kim J.H., Prameswati A., Park J., Aisyah S., Entifar N., Lee J., Kim S., Lim D.C. (2022). Multiple functionalities of highly conductive and flexible photo- and thermal-responsive colorimetric cellulose films. Mater. Res. Lett..

[34] Phan G.T., Pham D.V., Patil R.A., Tsai C.-H., Lai C.-C., Yeh W.-C., Liou Y., Ma Y.-R. (2021). Fast-switching electrochromic smart windows based on NiO-nanorods counter electrode. Sol. Energy Mater. Sol. Cells.

[35] Wang J.-L., Sheng S.-Z., He Z., Wang R., Pan Z., Zhao H.-Y., Liu J.-W., Yu S.-H. (2021). Self-Powered Flexible Electrochromic Smart Window. Nano Lett..

[36] Chen K., He J., Zhang D., You L., Li X., Wang H., Mei J. (2021). Bioinspired Dynamic Camouflage from Colloidal Nanocrystals Embedded Electrochromics. Nano Lett..

[37] Yu H., Qi M., Wang J., Yin Y., He Y., Meng H., Huang W. (2019). A feasible strategy for the fabrication of camouflage electrochromic fabric and unconventional devices. Electrochem. Commun..

[38] Sinha S., Daniels R., Yassin O., Baczkowski M., Tefferi M., Deshmukh A., Cao Y., Sotzing G. (2021). Electrochromic Fabric Displays from a Robust, Open-Air Fabrication Technique. Adv. Mater. Technol..

[39] Yu H., Shao S., Yan L., Meng H., He Y., Yao C., Xu P., Zhang X., Hu W., Huang W. (2016). Side-chain engineering of green color electrochromic polymer materials: Toward adaptive camouflage application. J. Mater. Chem. C.

[40] Gicevicius M., Cechanaviciute I.A., Ramanavicius A. (2020). Electrochromic Textile Composites Based on Polyaniline-Coated Metallized Conductive Fabrics. J. Electrochem. Soc..

[41] Li K., Zhang Q., Wang H., Li Y. (2014). Red, Green, Blue (RGB) Electrochromic Fibers for the New Smart Color Change Fabrics. ACS Appl. Mater. Interfaces.

[42] Fan H., Li K., Liu X., Xu K., Su Y., Hou C., Zhang Q., Li Y., Wang H. (2020). Continuously Processed, Long Electrochromic Fibers with Multi-Environmental Stability. ACS Appl. Mater. Interfaces.

[43] Gao X., Wang Y., Wu M., Zhi C., Meng J., Zhang L. (2023). Multicolor electrochromic fabric with a simple structure of PEDOT:PSS/DMSO. Dye. Pigment..

[44] Li M., Jiang W., Lin Y., Huang C., Hao P., Wang W., Yang L., Wang Y., Wang D. (2024). Preparation of WO3-based flexible electrochromic fabrics and their near infrared shielding application. J. Mater. Chem. C.

[45] Yang G., Fan J., Zhang K., Gu C., Li J., Kang K., Xiang C., Qian L., Zhang T. (2024). Electrochromic Reflective Displays Based on In Situ Photo-Crosslinked PEDOT: PSS Patterns. Adv. Funct. Mater..

[46] Liu L., Yang H., Zhang Z., Wang Y., Piao J., Dai Y., Cai B., Shen W., Cao K., Chen S. (2023). Photopatternable and Highly Conductive PEDOT:PSS Electrodes for Flexible Perovskite Light-Emitting Diodes. ACS Appl. Mater. Interfaces.

[47] Pinto C.S., Souza V.H., Schmidt A., Zarbin A.J. (2023). PSS-free PEDOT and PEDOT/graphene transparent films: Synthesis, characterization and electrochromism. Synth. Met..

[48] Wang Y., Pang F.F., Liu D.D., Han G.-Z. (2017). In situ synthesis of PEDOT: PSS@ AgNPs nanocomposites. Synth. Met..

[49] Huang L.M., Chen C.H., Wen T.C. (2006). Development and characterization of flexible electrochromic devices based on polyaniline and poly (3, 4-ethylenedioxythiophene)-poly (styrene sulfonic acid). Electrochim. Acta.

[50] (2022). Casual Wear.

[51] Fu G., Gong H., Xu J., Zhuang B., Rong B., Zhang Q., Chen X., Liu J., Wang H. (2024). Highly integrated all-in-one electrochromic fabrics for unmanned environmental adaptive camouflage. J. Mater. Chem. A.

[52] (2023). Test Method for Peeling Strength of Garments Used Adhesive Interlining.

